# Supplementary material for: Identification of BRCC3 and BRCA1 as Regulators of TAZ Stability and Activity
Source: Cells. 2023 Oct 11;12(20):2431. doi: 10.3390/cells12202431 (PMC10605050; doi:10.3390/cells12202431)
Supplement: Supplementary file 1 [file cells-12-02431-s001.zip › File S1. WB raw data.pdf]

FIG 2b

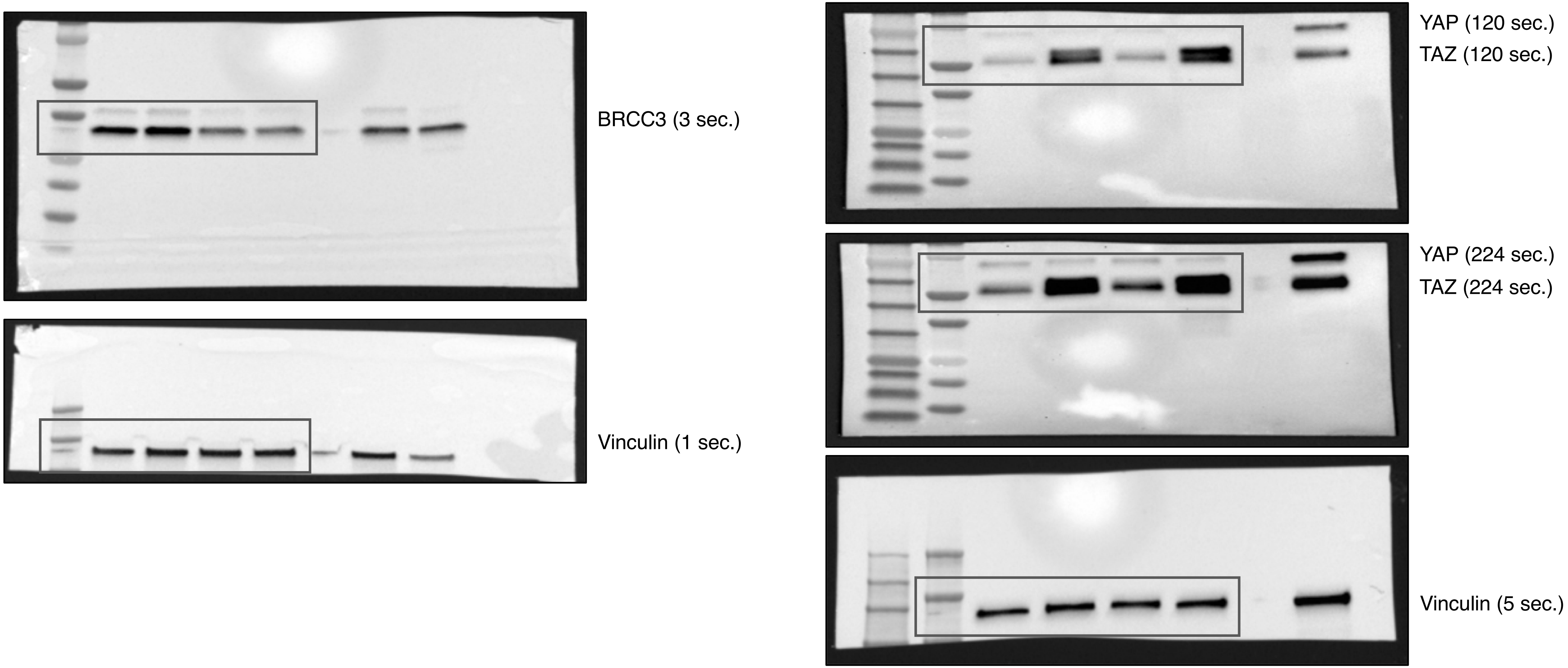
BRCC3 (3 sec.)
Vinculin (1 sec.)
YAP (120 sec.)
TAZ (120 sec.)
YAP (224 sec.)
TAZ (224 sec.)
Vinculin (5 sec.)

FIG 2d

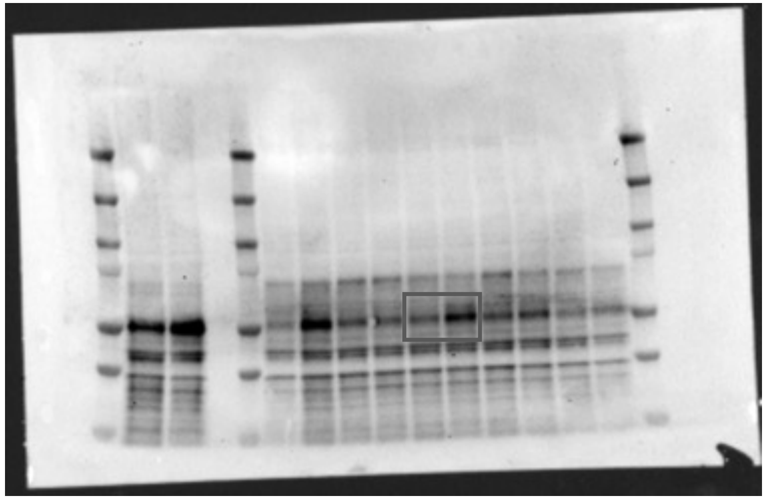

TAZ (151,3 sec.)

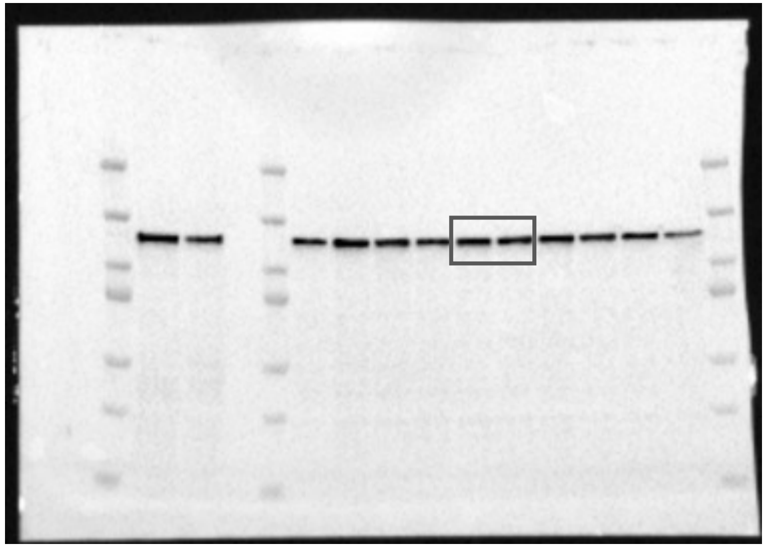

Vinculin (1 sec.)

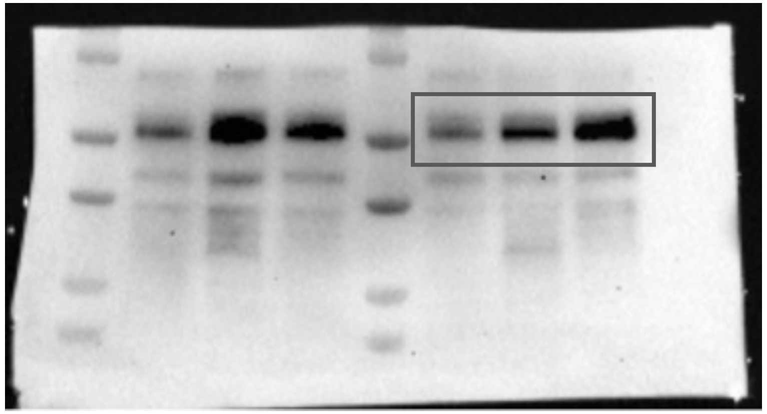

TAZ (173,6 sec.)

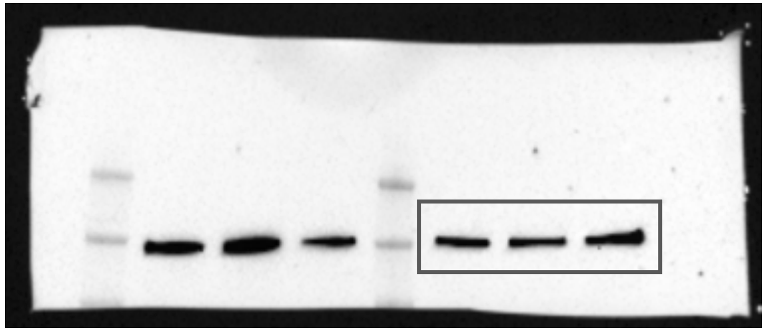

Vinculin (200 sec.)

FIG 2e

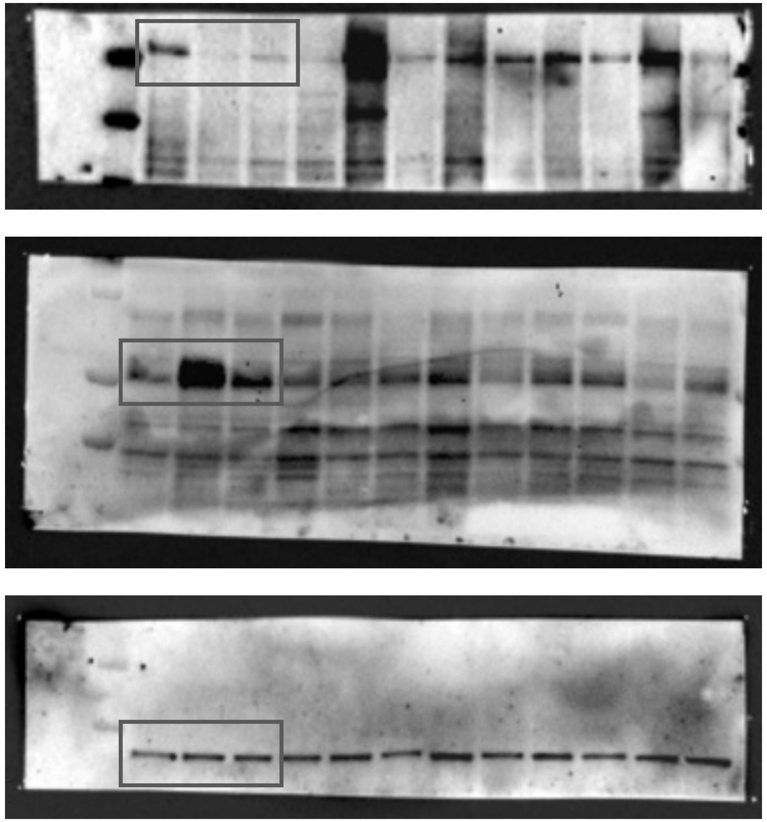

BRCA1 (408,3 sec.)

TAZ (205,6 sec.)

Vinculin (4 sec.)

FIG 2f

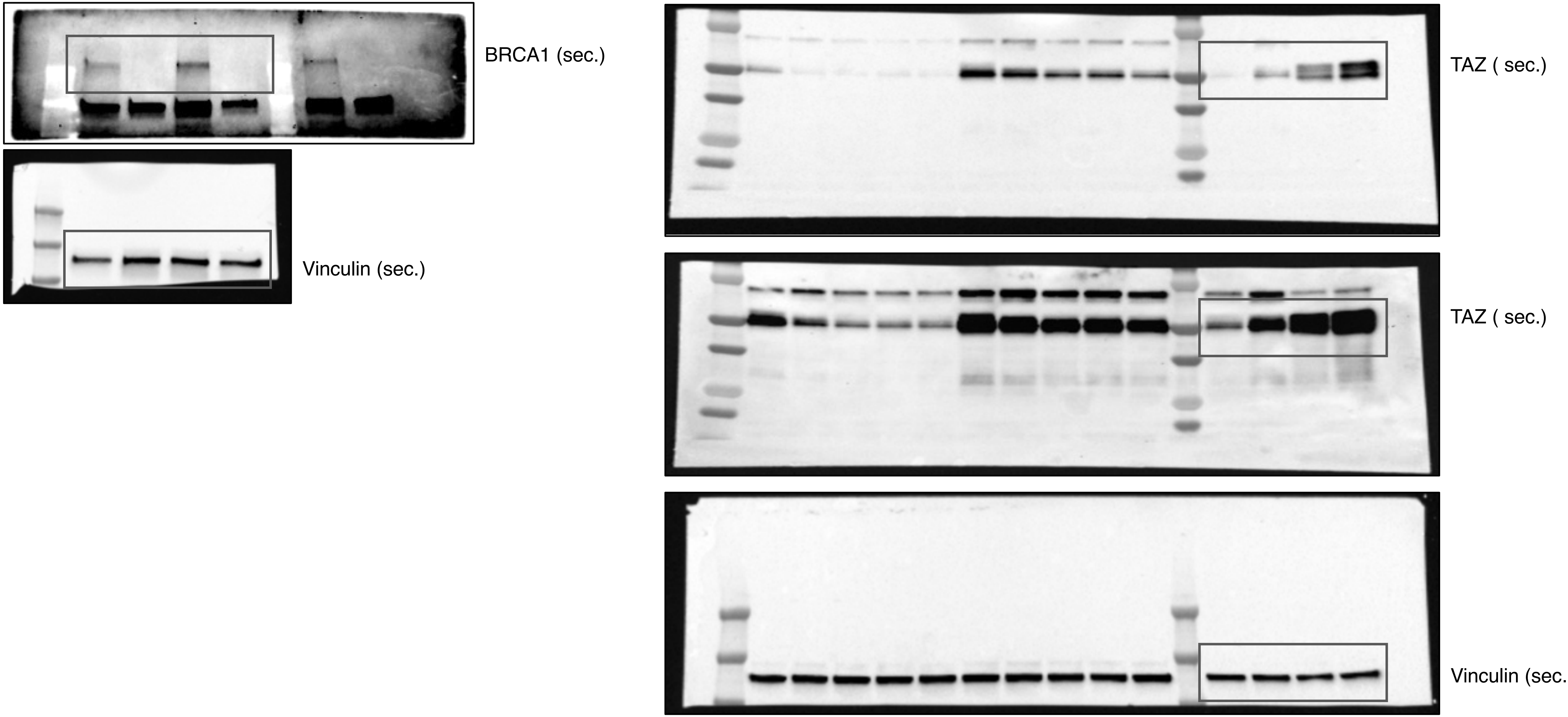

FIG 2g

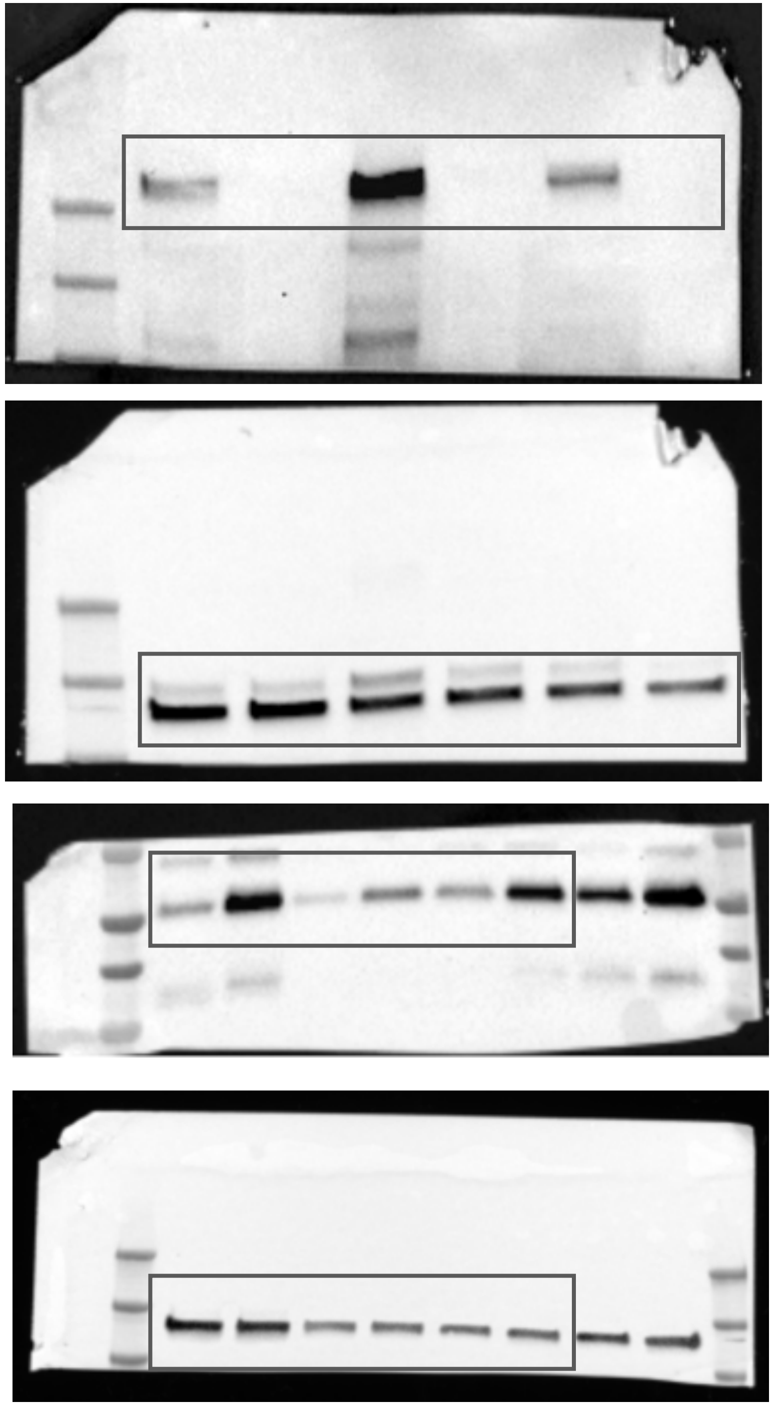

FIG 5a

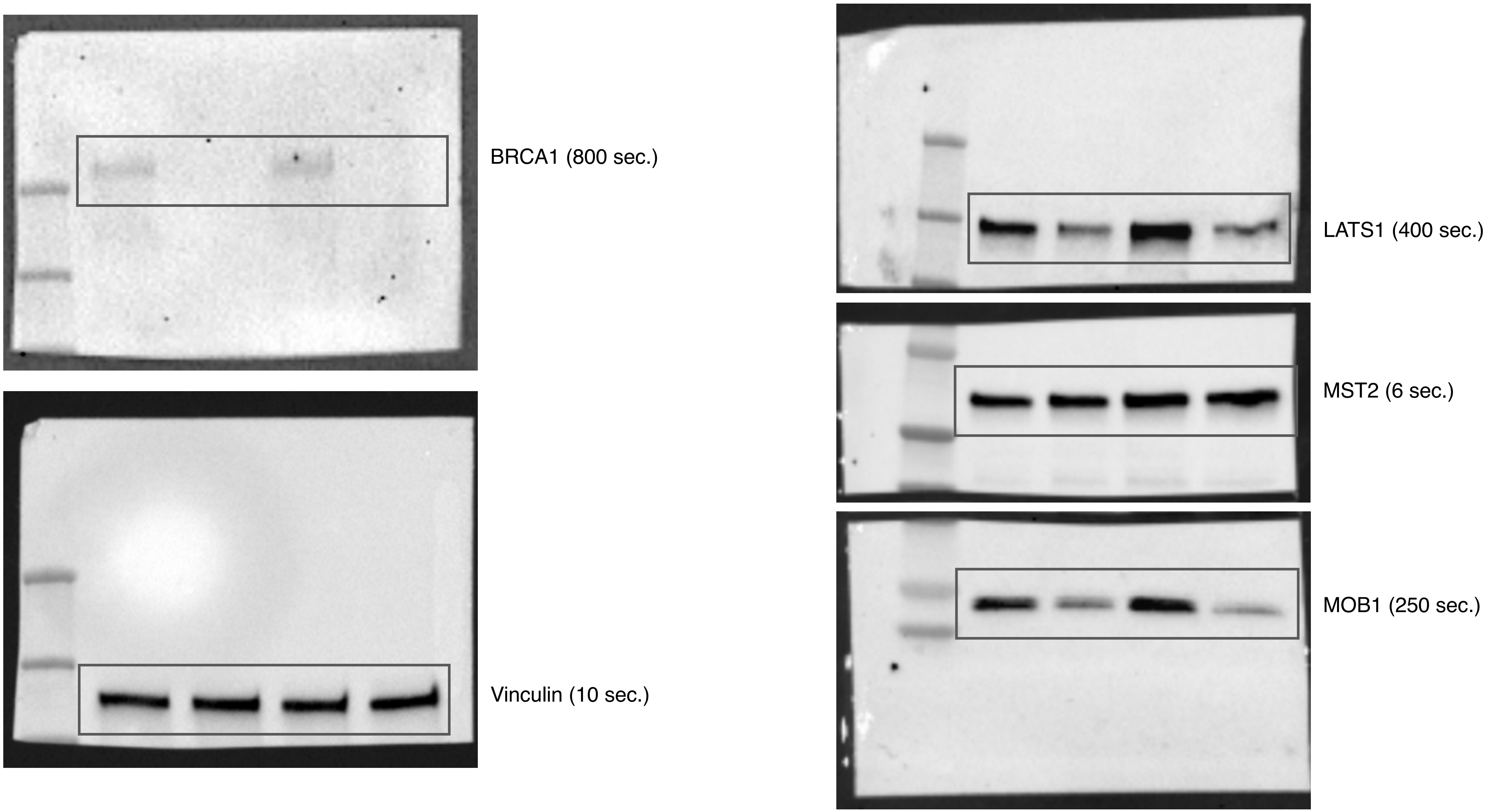
BRCA1 (800 sec.)
Vinculin (10 sec.)
LATS1 (400 sec.)
MST2 (6 sec.)
MOB1 (250 sec.)

FIG 5b

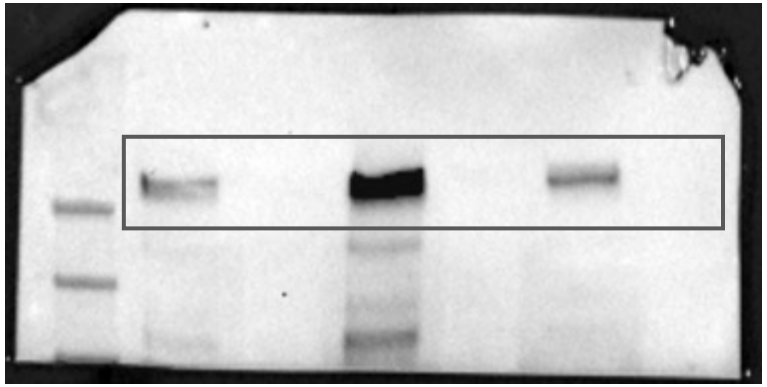

BRCA1 (300 sec.)

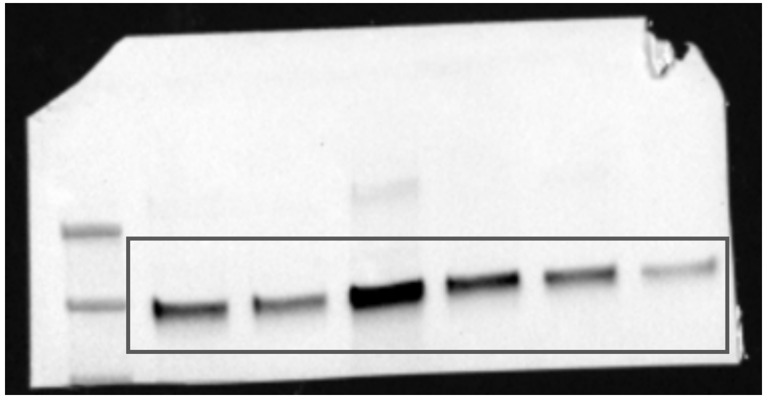

LATS1 (50 sec.)

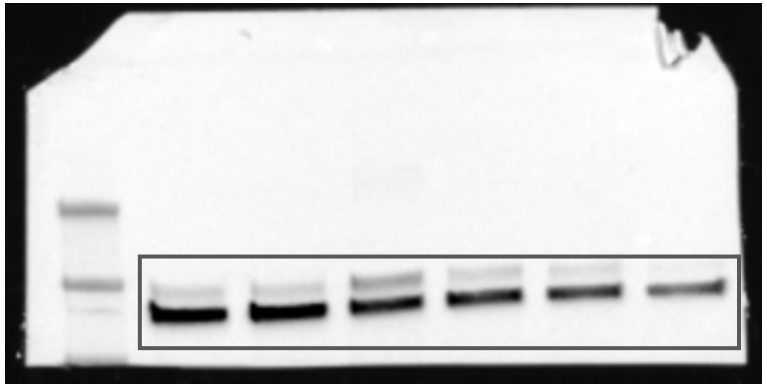

Vinculin (13 sec.)

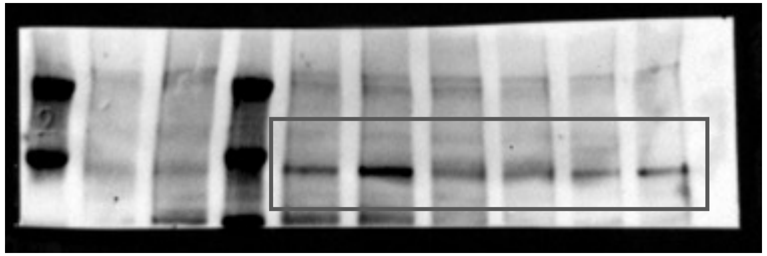

LATS2 (141,3 sec.)

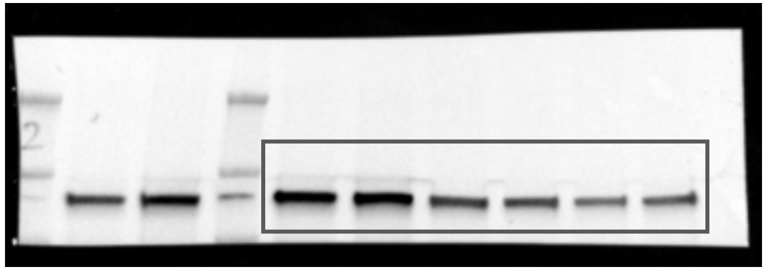

Vinculin (3,6 sec.)

FIG 5c

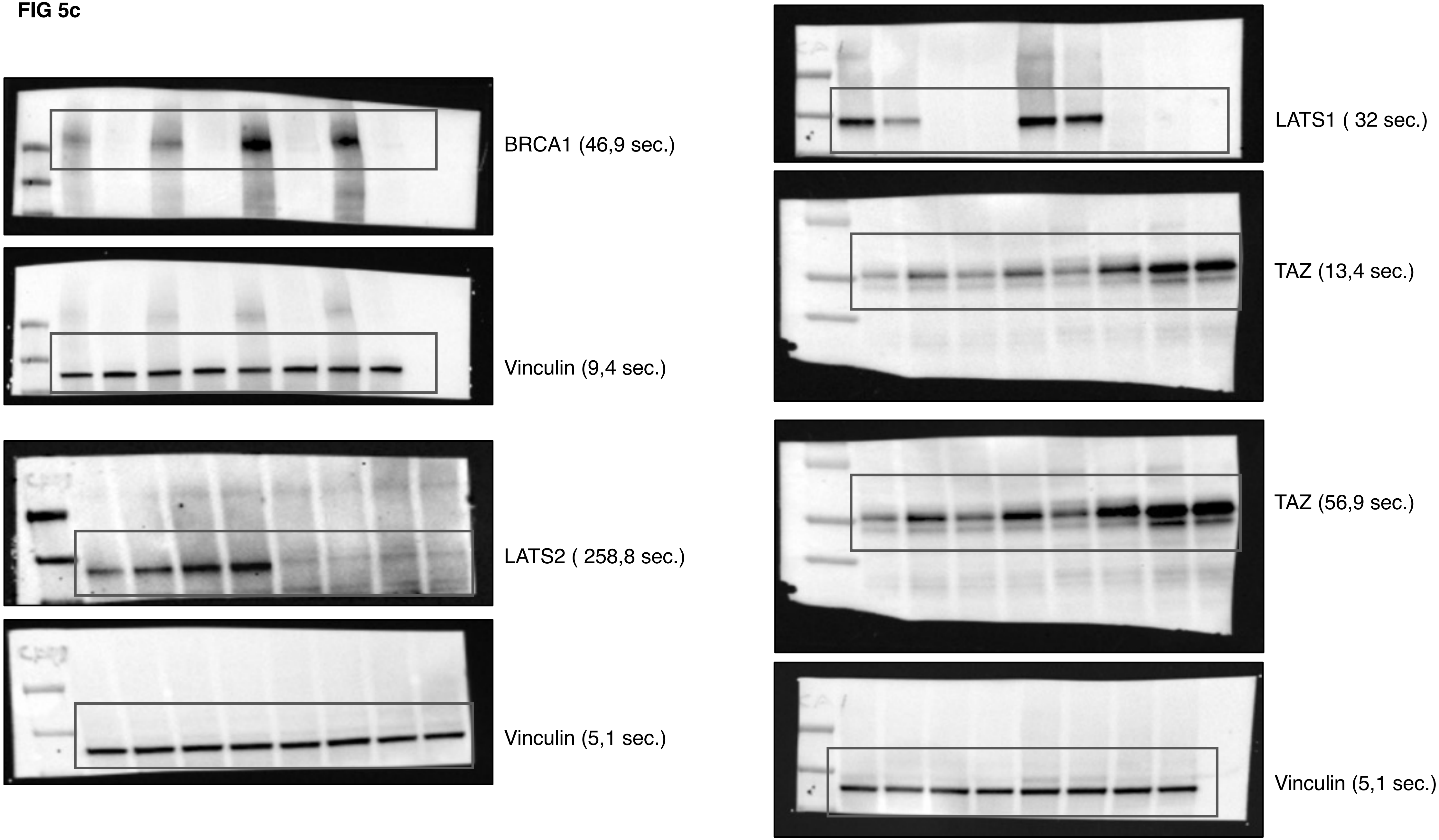
BRCA1 (46,9 sec.)
Vinculin (9,4 sec.)
LATS2 (258,8 sec.)
Vinculin (5,1 sec.)
LATS1 (32 sec.)
TAZ (13,4 sec.)
TAZ (56,9 sec.)
Vinculin (5,1 sec.)

FIG 5d

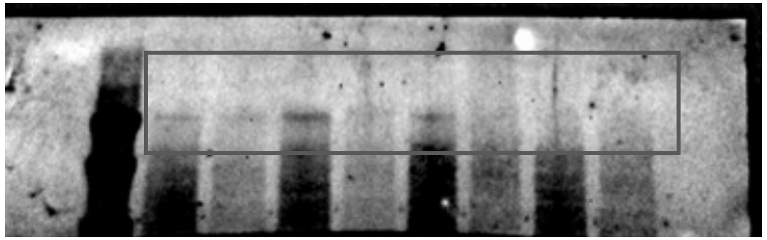

BRCA1 (200 sec.)

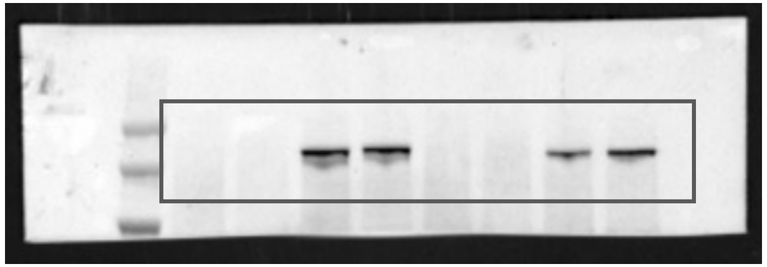

LATS1 (26,1 sec.)

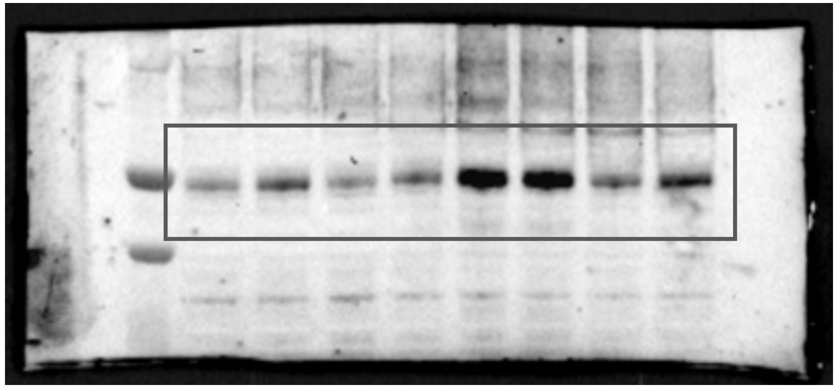

TAZ (84,4 sec.)

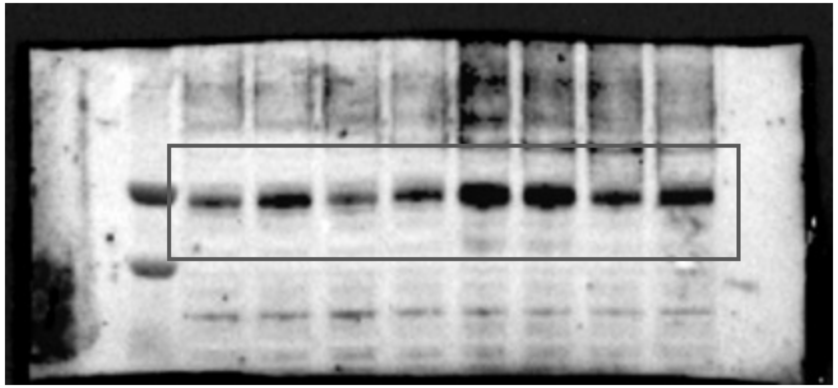

TAZ (100 sec.)

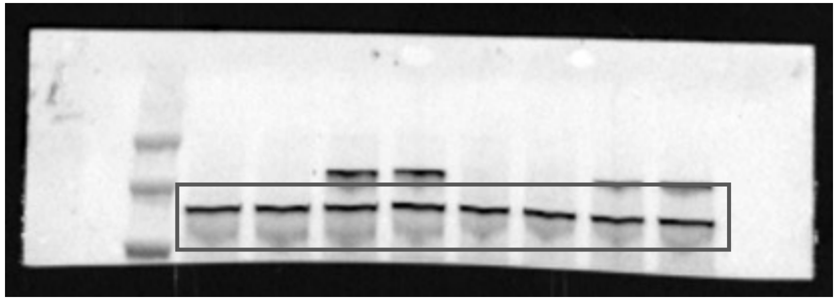

Vinculin (16,5 sec.)

FIG 6d

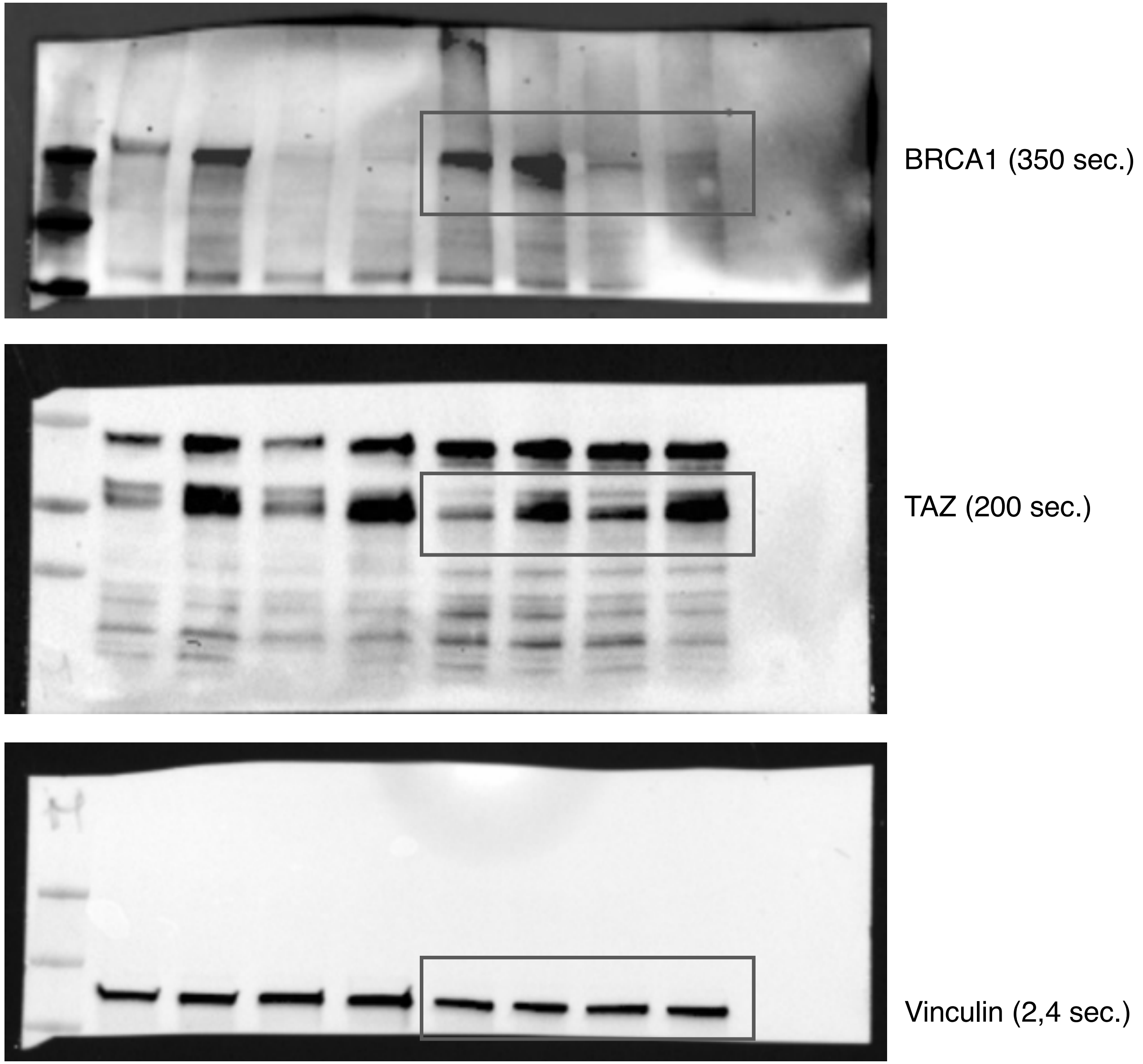

FIG 7a

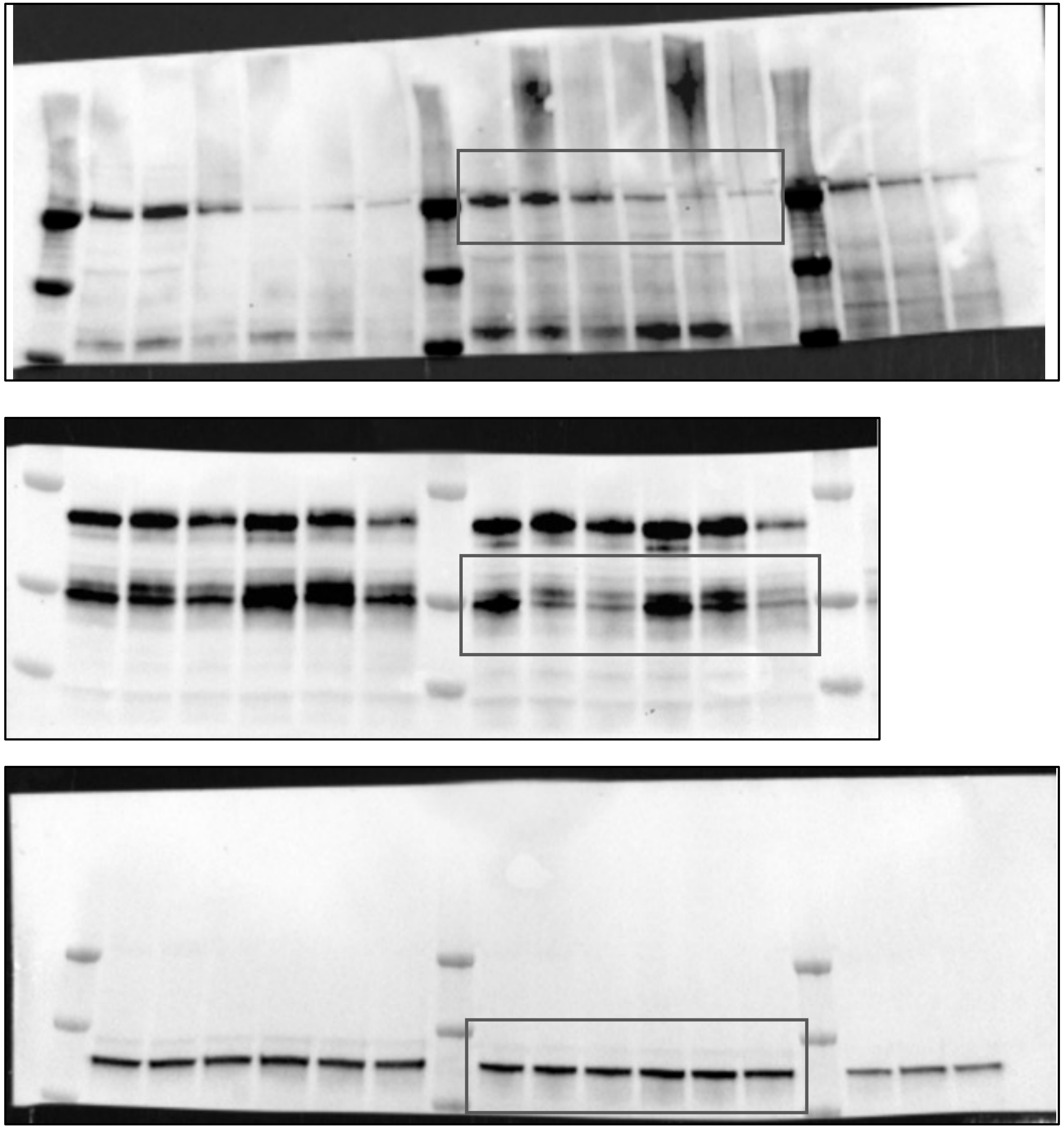

FIG 7c

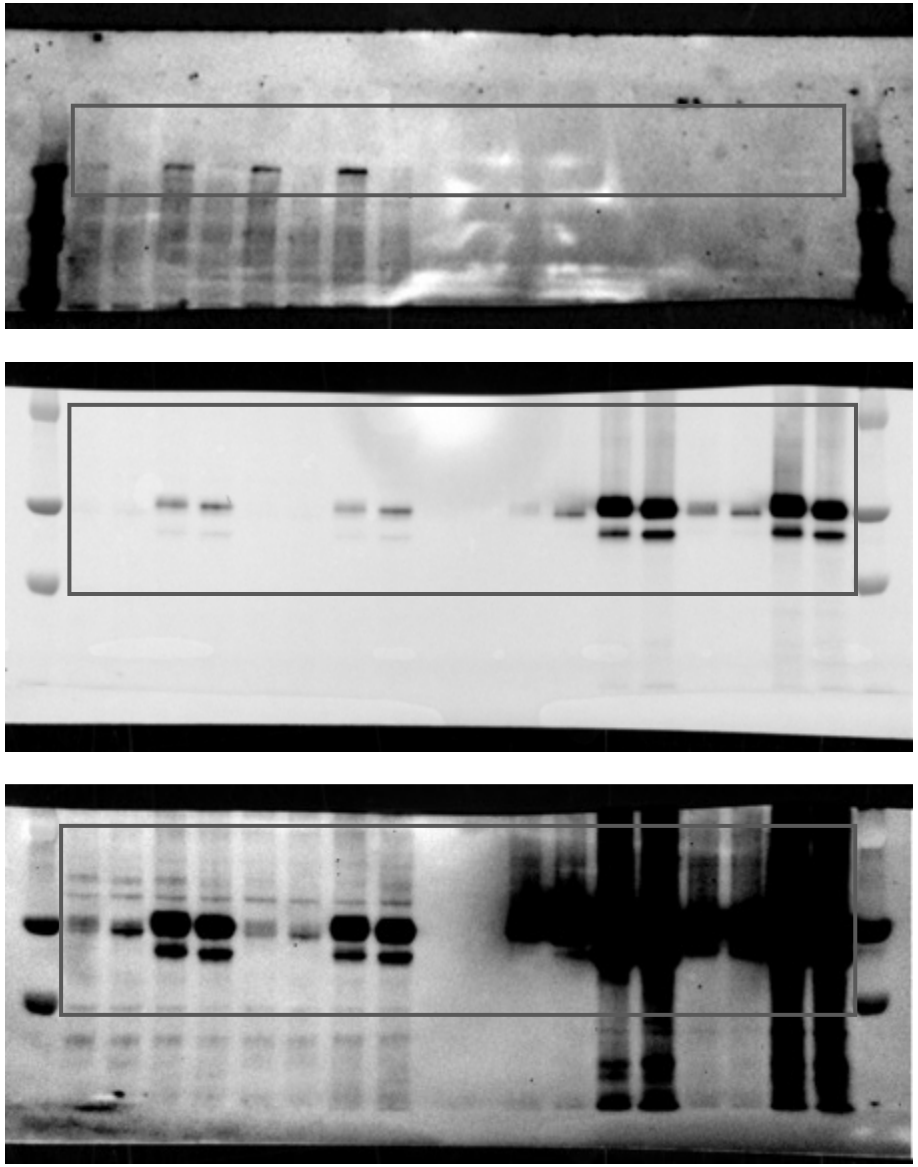

BRCA1 (269,7 sec.)

TAZ (21,3 sec.)

TAZ (339,8 sec.)

FIG 7d

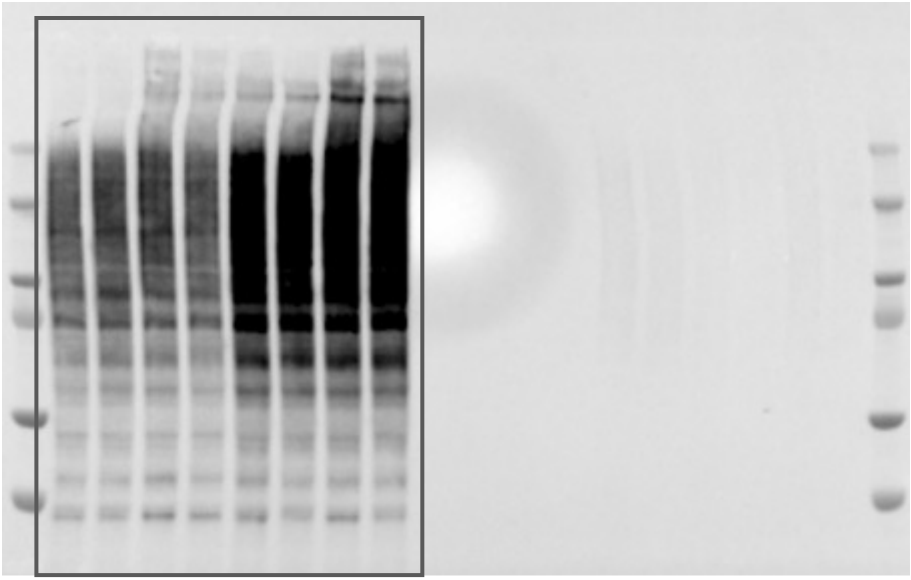

Ub (1 sec.)

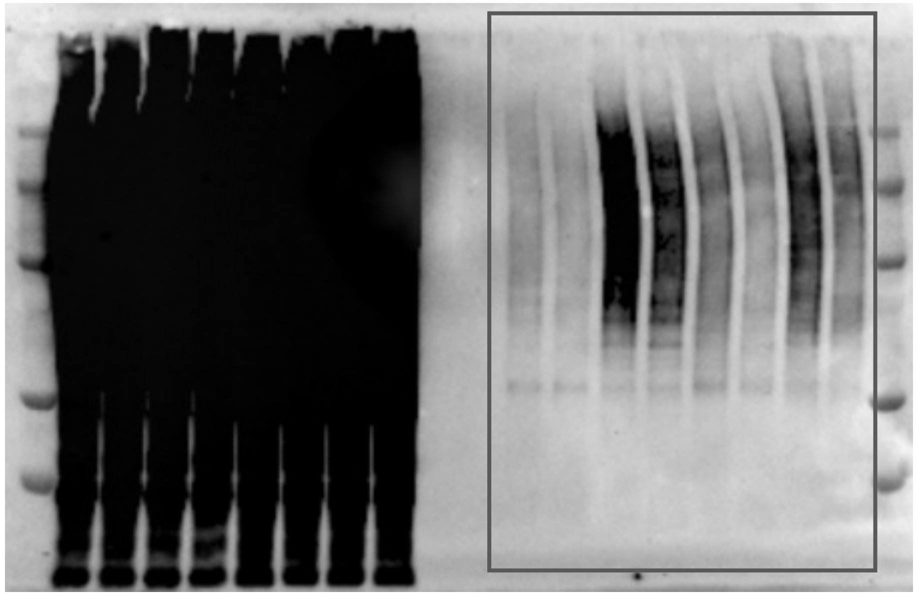

Ub (41,7 sec.)

FIG S2

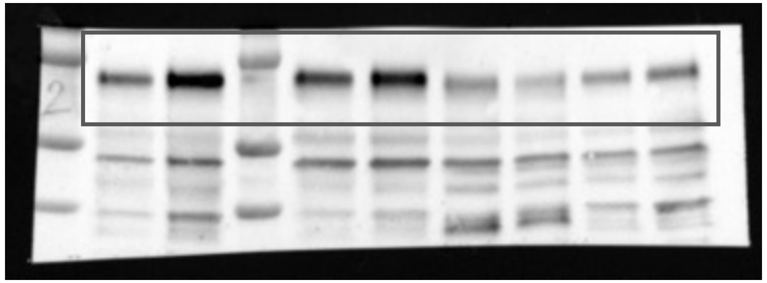

YAP (19,3 sec.)

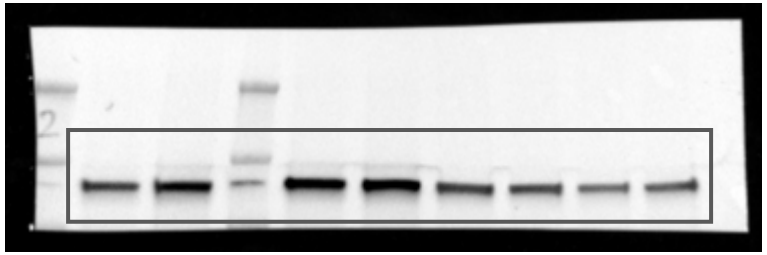

Vinculin (3,6 sec.)

FIG S3

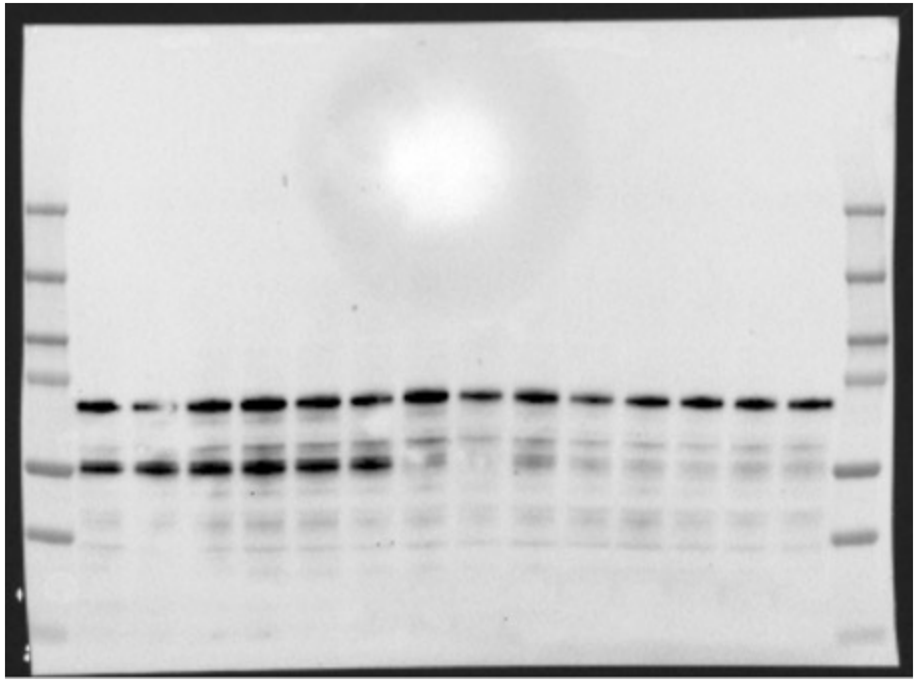

TAZ (171,4 sec.)

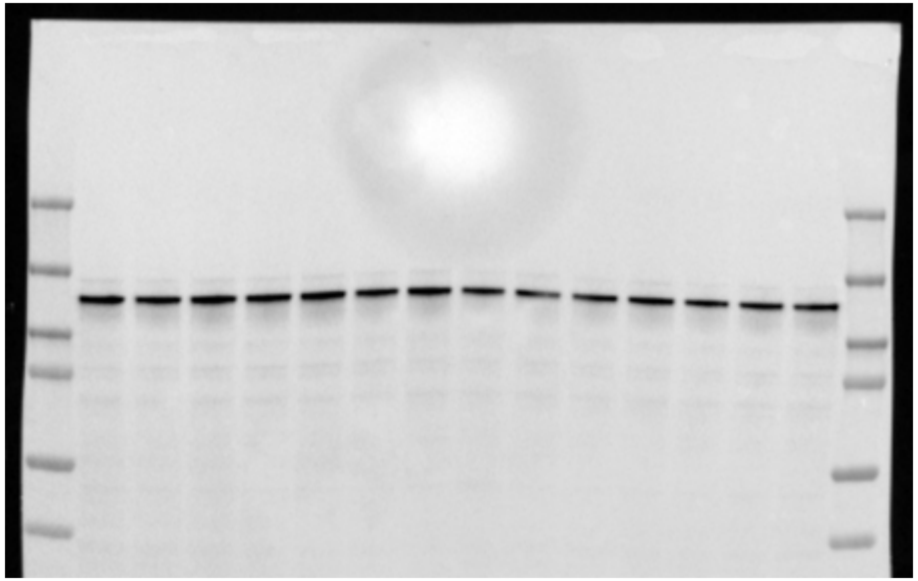

Vinculin (2,4 sec.)
