# Supplementary material for: Identification of BRCC3 and BRCA1 as Regulators of TAZ Stability and Activity
Source: Cells. 2023 Oct 11;12(20):2431. doi: 10.3390/cells12202431 (PMC10605050; doi:10.3390/cells12202431)
Supplement: Supplementary file 1 [file cells-12-02431-s001.zip › supplementary_figures.pdf]

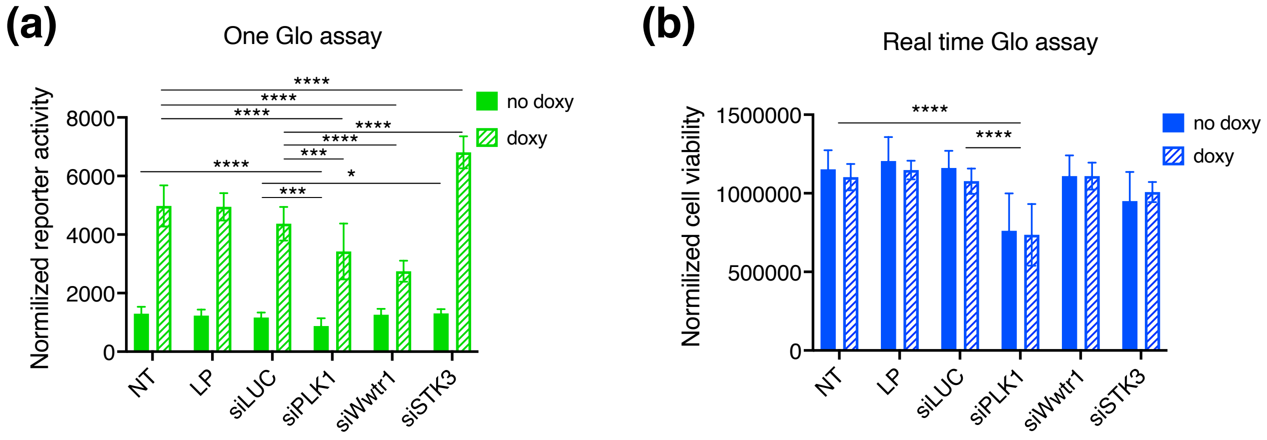

Figure S1. Assessment of the dynamic range of the assays used for the siRNA screen.
(a,b) Bar plots showing (a) the normalized reporter activity (one glo assay) and (b) cell viability (real time glo assay) in MCF10A-pSLIK-TAZS89A-8xTEAD-Luc transfected with the negative control siLUC, the siPLK1 (essential gene), siWwtr1 and siSTK3 (respectively a negative and positive control for TAZ activity). NT refers to not-transfected cells, while LP refers to cells transfected with lipofectamine but without any siRNA. T-test was applied to evaluate the statistical significance: * p value < 0.05, *** p value < 0.005, **** p value < 0.001.

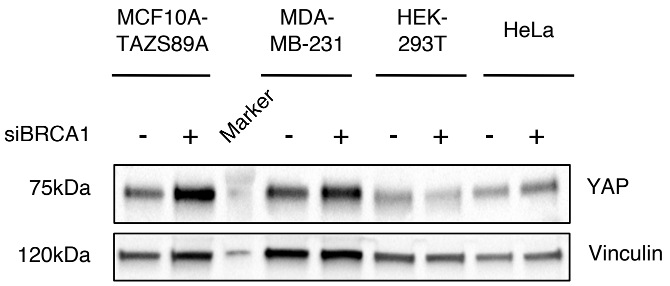

Figure S2. Inconsistent regulation of YAP1 by BRCA1 in different cell lines.
WB analysis showing YAP protein levels upon BRCA1 knock-down in multiple cell lines. Cells were transfected with siBRCA1 (#458) or with a non-targeting siRNA (siC) as control. After 48 hours sub-confluent cells were lysed for protein extraction and WB analysis. Vinculin was used for loading control.

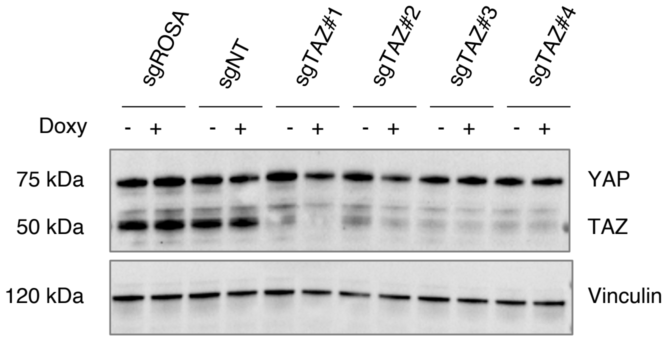

Figure S3. WB analysis of MCF10A-tet-Cas9 cells expressing non targeting sgRNA (sgROSA, sgNT) and sgRNAs targeting TAZ (sgTAZ). Doxycycline (doxy) was used to induce the Cas9. Please note that, due to the leakiness of the Cas9 expression, TAZ expression is lost also in mock cells (no doxycycline).
